# Sepsis-induced modulation of long-term potentiation induced by theta burst stimulation in the rat hippocampus

**DOI:** 10.3389/fnins.2023.1296391

**Published:** 2023-11-24

**Authors:** Ryuichiro Kakizaki, Eichi Narimatsu, Takehiko Kasai, Kazuhito Nomura

**Affiliations:** Department of Emergency Medicine, Sapporo Medical University, Sapporo, Japan

**Keywords:** synaptic transmission, hippocampus, long-term potentiation, theta burst stimulation, sepsis-associated encephalopathy, superoxide dismutase

## Abstract

We investigated the influences of sepsis on central synaptic plasticity *in vitro*. Cecal ligation and puncture (CLP) was performed by creating rat sepsis models, which were divided into early and late sepsis groups (8 and 16 h after CLP, respectively). In the CA1 of the rat hippocampal slices, orthodromically elicited population spikes (PSs) and field excitatory postsynaptic potentials (fEPSPs) were simultaneously recorded, and their long-term potentiation (LTP) was induced by theta burst stimulation (TBS). TBS induced LTPs of PSs and fEPSPs in all groups. In the sham and early sepsis groups, there was no significant difference in LTPs between PSs and fEPSPs. However, in the late sepsis group, the LTP of PSs was greater than that of fEPSPs (*p* < 0.05) and was greater than the LTPs of PSs in the sham and early sepsis groups (*p* < 0.05). Superoxide dismutase, administered immediately before CLP, inhibited the enhancement of LTP in PS, as observed in the late sepsis group. The initial rapid potentiation component of LTP in fEPSPs was suppressed or reduced in all groups that underwent CLP. The results indicate that CLP-induced sepsis modulates hippocampal synaptic plasticity, depressing excitatory synaptic transmissions and facilitating somatic excitability, which is induced by septic oxygen superoxide.

## 1 Introduction

Sepsis is a complex disorder that develops as a dysregulated host response to an infection and is associated with acute organ dysfunction and high risk. Sepsis has a high incidence and remains one of the leading causes of death worldwide (Rudd et al., 2020).

Sepsis-associated encephalopathy (SAE), which commonly accompanies severe systemic infection, is defined as a diffuse cerebral dysfunction in the absence of a direct central nervous system (CNS) infection, structural abnormalities, or other types of encephalopathy (Iacobone et al., 2009; Gofton and Young, 2012). Neurological dysfunction during SAE ranges from mild confusion and lethargy to disturbed cognitive functions and coma (Ebersoldt et al., 2007). Although SAE is considered a reversible syndrome, mild to moderate neurological symptoms, including memory alterations, depression, anxiety, and cognitive disturbances, may persist in up to 40% of patients one year after hospital discharge (Semmler et al., 2013). Memory impairment, in particular, has probably originated from dysfunction of the hippocampus (Squire and Zola-Morgan, 1991). It has been reported that these long-term cognitive alterations are caused by neurodegenerative microglial activation and diffuse ischemic damage, producing the same histopathological changes as chronic neurodegenerative diseases (Gunther et al., 2012; Wang et al., 2018). Moreover, sepsis is related to long-lasting myeloid inflammation of the CNS in mouse models induced by cecal ligation and puncture (CLP) (Singer et al., 2016).

The pathophysiology of SAE is poorly understood, and SAE is thought to be caused by one or a combination of the following conditions: abnormal neurotransmission (Li et al., 2022), activation of the vascular endothelium by inflammatory cytokines, disruption of the blood–brain barrier (Esen et al., 2005), brain mitochondrial dysfunction (Bozza et al., 2013), and impaired microcirculation in the brain (Kafa et al., 2007). The abnormalities in neurotransmission may have originated from imbalances in amino acid levels (Basler et al., 2002; Berg et al., 2010). Few studies have verified the electrophysiological aspects of central synaptic plasticity in sepsis; however, sepsis-induced increase in transmitter release and decrease in critical depolarization on the postsynaptic membrane have been reported in peripheral motor synapse (Niiya et al., 2006).

In addition, the brain is constantly exposed to relatively high levels of reactive oxygen species (ROS), primarily because of the rapid rate of oxygen metabolism and the leakage of superoxide anions from the mitochondrial electron transport chain (Halliwell, 2006). Increased superoxide dismutase (SOD) levels have been reported in the brains of septic rats (Barichello et al., 2006). Thus, ROS is presumed to be associated with altered synaptic plasticity during sepsis; however, the details have not been clarified.

This study was designed to directly test the hypothesis that sepsis modulates the plasticity of excitatory synaptic transmissions in the CNS mediated by ROS. We investigated the influences of septic conditions on the long-term potentiation (LTP) of population spikes (PSs) and field excitatory postsynaptic potentials (fEPSPs) recorded from the CA1 region of hippocampal slices and the effects of SOD on the influences of sepsis. PSs and fEPSPs mainly reflect action potentials at the somas and glutamatergic excitatory postsynaptic potentials (EPSPs) at the dendrites of CA1-pyramidal neurons, respectively (Richardson et al., 1987).

## 2 Materials and methods

All experimental protocols were approved by the Animal Use Committee of Sapporo Medical University. Male Sprague–Dawley (SD) rats (4–5 weeks and weighing 150–180 g) were used in this study. To induce experimental sepsis, CLP was performed. CLP animals exhibit hyperdynamic, hypermetabolic sepsis in the early phase (within 10 h after CLP), followed by hypodynamic, hypometabolic sepsis in the late phase (12–30 h after CLP) (Wichterman et al., 1980). Therefore, we defined the early and late sepsis phases as 8 and 16 h after CLP operations, respectively. It has been reported that experimental septic phases at 8 and 16 h after CLP operations were equivalent for clinical early and late sepsis, respectively (Chaudry et al., 1979; Chen et al., 1994).

The rats were randomly divided into the following five groups:

a) Normal group: no intervention was performed before brain removal.
b) Sham group: only a sham laparotomy was done before brain removal.
c) CLP-8 h group, a CLP operation was performed 8 h before brain removal.
d) CLP-16 h group, a CLP operation was performed 16 h before brain removal.
e) CLP-16 h-SOD group: PEG-SOD was administered 30 min before the CLP operation, and the brain was removed 16 h after the CLP operation.

### 2.1 Animals and sepsis induction protocol

SD rats were anesthetized with isoflurane, and subsequently, a 1–2 cm midline incision was made in the abdominal wall.

The incision was closed without touching the intestinal tract (sham group). CLP operation: The cecum was exposed and tightly ligated just below the ileocecal valve using a 2–0 silk suture, avoiding bowel obstruction. The cecum was then punctured twice from side to side with a 19-gauge needle. The cecum was gently squeezed until feces were visible through the punctures and then placed again in the abdominal cavity. Thereafter, the incision was closed, and antibiotics were not administered to the rats. All rats were resuscitated with saline solution (5 mL/100 g body weight), which was administered subcutaneously to their backs at the time of the operation. The rats were deprived of food but had free access to water postoperatively in the sham, CLP-8 h, CLP-16 h, and CLP-16 h-SOD groups. Superoxide dismutase (SOD) was used as an oxygen-free radical scavenger. PEG-SOD (4,000 U/kg, Sigma, St. Louis, MO, United States), dissolved in saline (2 mL), was administered intraperitoneally 30 min before CLP in the CLP-SOD group.

### 2.2 Preparation of hippocampal slices

Rats were deeply anesthetized with isoflurane, the brains were rapidly removed from each rat, and 6–8 transverse hippocampal slices (400 μm in thickness) were prepared from each hemisphere with a vibratome (Leica VT 1000S; Leica, Nusslcoch, Germany) in sucrose-based artificial cerebrospinal fluid (S-ACSF) oxygenated with 95% O_2_–5% CO_2_ gas at 3–4°C (pH 7.40 ± 0.05). The S-ACSF composition was as follows (mM): sucrose, 220; KCl, 3.0; CaCl_2_, 2.0; MgSO_4_, 2.0; NaHCO_3_, 26.0; Na_2_HPO_4_, 1.25; and glucose, 10. Slices were then incubated for at least 1 h in oxygenated normal artificial cerebrospinal fluid (ACSF) at 34°C. The ACSF composition was as follows (in mM): NaCl, 123.0; KCl, 4.5; CaCl_2_, 2.5; MgSO_4_, 1.2; NaHCO_3_, 25.0; Na_2_HPO_4_, 1.2; and glucose, 10.

### 2.3 Measurements

**Figure 1:**
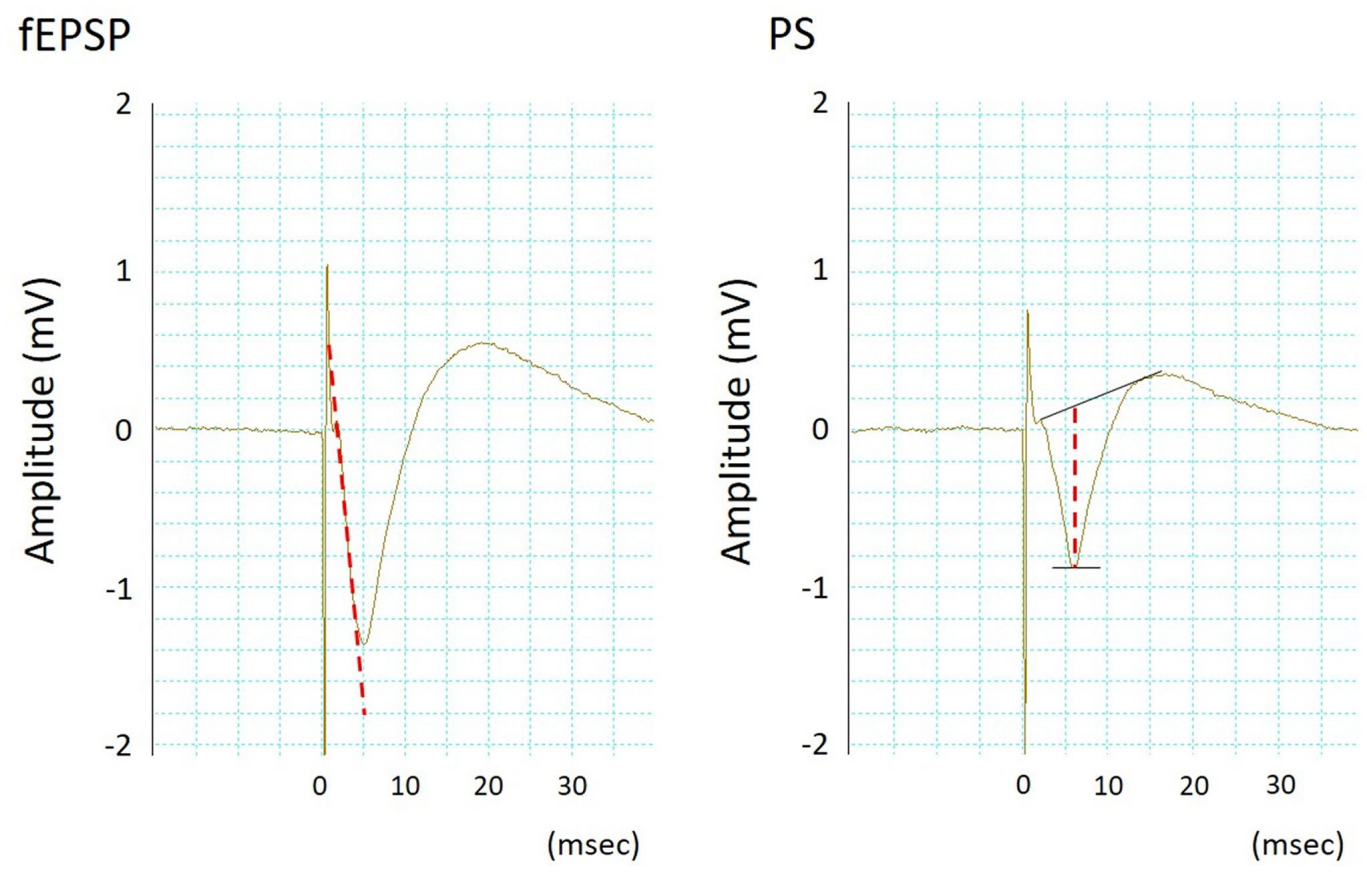
Typical waveforms of simultaneously recorded field excitatory postsynaptic potentials (fEPSPs, left) and population spikes (PSs, right). The fEPSP slope and PS amplitude were measured according to the dotted lines.

PSs and fEPSPs were simultaneously recorded using an MEA system (Multi Channel Systems, Reutlingen, Germany), a multichannel recording system. The slices were placed in a submerged multi-electrode array (MEA) chamber (1 mL, vol.) and constantly superfused (4 mL/min) with ACSF oxygenated with 95% O_2_–5% CO_2_ at 34.0 ± 0.2°C (pH 7.40 ± 0.05). A total of 60 titanium nitride-plate microelectrodes (diameter: 0.03 mm, resistance <50 kΩ) were arranged in a square-latticed pattern (inter-electrode distance: 0.2 mm) on the base of the MEA chamber. Two side-by-side microelectrodes positioned at the CA1-stratum pyramidale and the CA1-stratum radiatum that corresponded to the somas and dendrite concentrations in a group of pyramidal cells, respectively, were chosen for recording PSs and fEPSPs. The PSs and fEPSPs signals from the microelectrodes were amplified and filtered (bandpass, 10 Hz–3 kHz) using an MEA 1060 amplifier (Multi Channel Systems) and recorded using PowerLab 16/30 (AD Instruments Bella Vista, Australia). PSs and fEPSPs were elicited by orthodromic electrical stimulation on Schäffer collaterals [biphasic negative–positive current steps; 0.1 ms/phase, 0.1 Hz, 0.2 mA, using a computer-controlled stimulator (STG 4002, Multi Channel Systems)]. Two side-by-side microelectrodes positioned at the CA1-stratum radiatum/moleculare, where Schäffer collaterals pass and 0.2 mm from the CA1-stratum radiatum for fEPSP recording, were used as bipolar stimulating electrodes. The desired orientation of the hippocampal area with respect to the microelectrodes was achieved by precisely positioning the slices using a fine nylon brush and a microscope. As indicators of synaptic strength at the dendrites and somatic excitability, fEPSP slope and PS amplitude, respectively, averaged over six groups (Figure 1), were analyzed online using a computer program in PowerLab 16/30.

### 2.4 Study design of electrophysiology

Simultaneous PSs and fEPSPs recordings in normal ACSF for a control scan of 10 min were initiated after stabilization, which was confirmed when the variability of the PS amplitude and fEPSP slope were within the range of 5% of the mean value for a period of more than 10 min. Theta-burst stimulation (TBS; 15 trains of four pulses at 100 Hz were delivered at 5 Hz) was performed, inducing long-term potentiation (LTP). LTP is recorded for 60 min after induction. PS and fEPSP were recorded by researchers who were blinded to the experimental group to which each hippocampal slice belonged.

### 2.5 Statistics

Data are expressed as the means ± standard deviation (% of the control). Control values were defined as the mean PS amplitude and mean fEPSP slope in the last 1 min immediately before TBS. Initial rapid potentiation (IRP) of LTP was defined as the mean PS amplitude and mean fEPSP slope at the first 1 min immediately after TBS. One-or two-way repeated-measures analysis of variance (ANOVA), one-way factorial ANOVA, and Bonferroni/Dunn analysis for *post-hoc* testing were used for statistical comparison, and *p* < 0.05 was set as the significance threshold.

## 3 Results

In the CLP-16 h group, rats demonstrated decreased spontaneous activities and escaping movements, napped hair before dissection, and showed severe inflammatory changes in systemic organs (severe panperitonitis; inflammatory ascites and edema of the intestines, liver, kidneys, lungs, peritoneum, etc.) during gross inspection upon dissection. These findings of panperitonitis in the CLP-8 h and CLP-16 h-SOD groups were less severe than those in the CLP-16 h group. Rats in the sham group did not show findings of panperitonitis.

Stimulations of Schäffer collaterals elicited PSs (0.19–2.41 mV in amplitude) and fEPSPs (0.073–0.527 mV/ms in slope) simultaneously. There was no significant difference in PS amplitude or fEPSP slope among these experimental groups. In time-control estimations in normal ACSF using normal rats, PS amplitude and fEPSP slope remained stable for at least 90 min after their stabilizations (95.4 ± 6.6% and 98.3 ± 13.9% of the control at 90 min, respectively, *n* = 6), which was sufficient to complete each experimental protocol.

### 3.1 LTP as normal and sham groups

**Figure 2:**
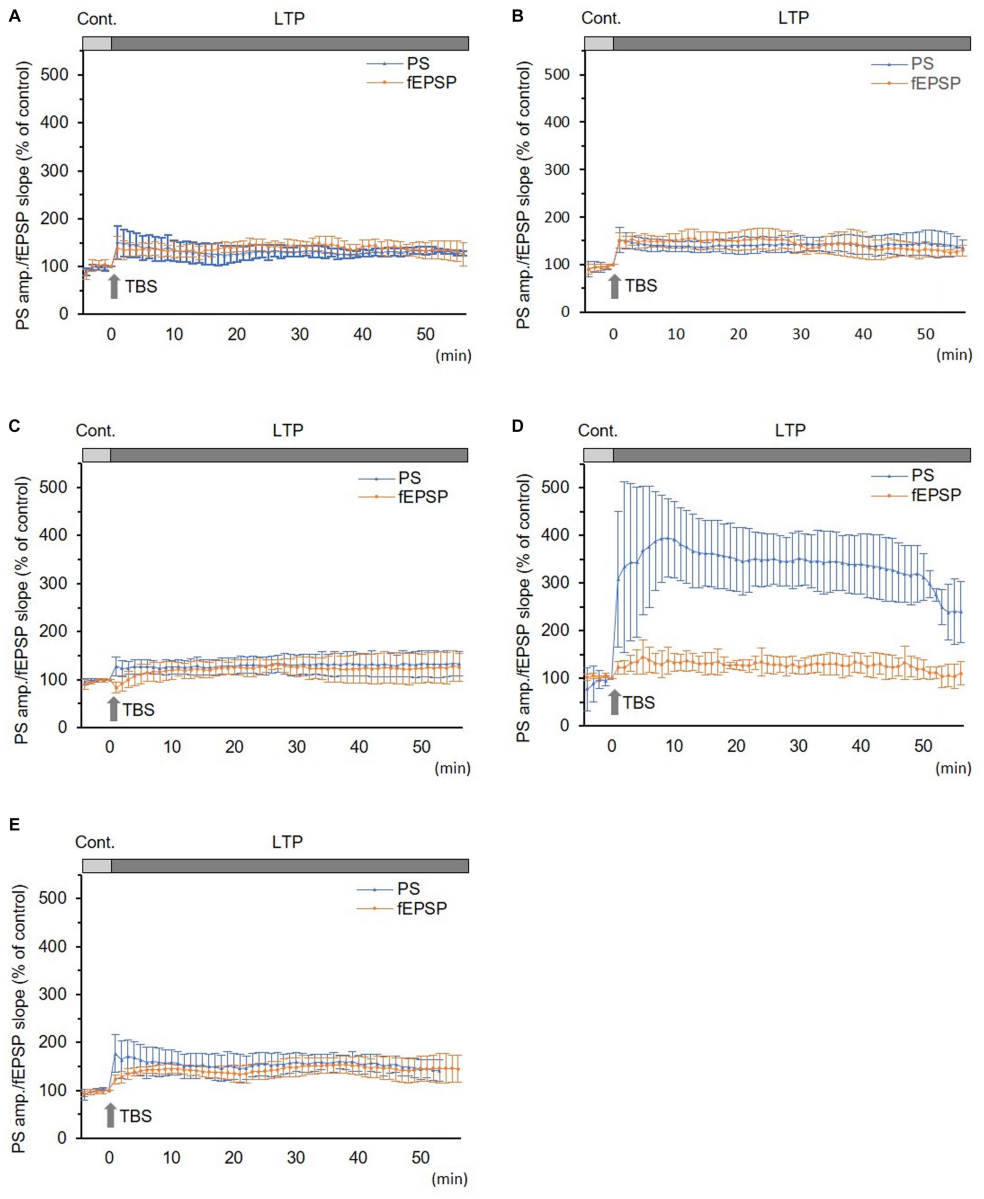
Long-term potentiation (LTP) of simultaneously recorded population spikes (PSs) and field excitatory postsynaptic potentials (fEPSPs). **(A)** Normal group (*n* = 6): there was no significant difference between LTPs in PSs and fEPSPs and between their initial rapid potentiations (IRPs). **(B)** Sham group (*n* = 5): there was no significant difference between LTPs in PSs and fEPSPs and between their IRPs. **(C)** CLP-8 h group (*n* = 5): there was no significant difference between LTPs in PSs and fEPSPs. IRPs in PSs were obvious; however, that in fEPSPs were not seen and rather suppressed; thereafter, LTP developed gradually. **(D)** CLP-16 h group (*n* = 5): LTP in PSs was significantly larger than that in fEPSPs, and its peak was delayed. LTP in fEPSPs developed gradually. **(E)** In the CLP-SOD group (*n* = 5), there was no significant difference between LTPs in PSs and fEPSPs. IRPs in PSs were significantly larger than those in fEPSPs. Data are expressed as mean ± standard deviation and % of the control value.

**Figure 3:**
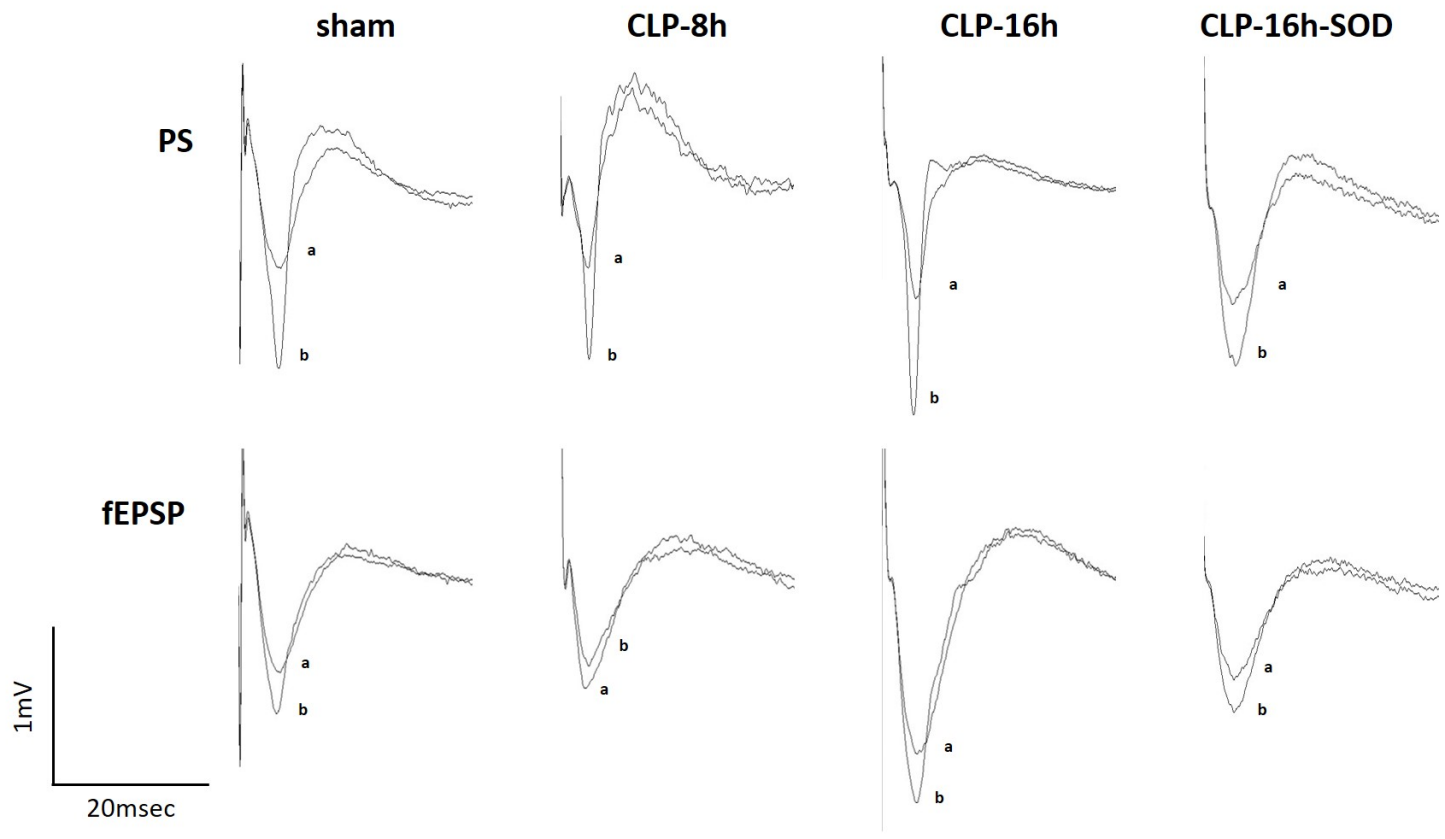
Representative changes in waveforms of population spikes (PSs, upper) and field excitatory postsynaptic potentials (fEPSPs, lower) between the control (a) and initial rapid potentiation (b).

In normal and sham groups, TBS-induced LTPs in PSs and fEPSPs (*p* < 0.05, Figures 2A,B). IRP in the normal group (150.5 ± 34.8% and 138.7 ± 23.8% of the control in PS amplitude and fEPSP slope, respectively, *n* = 6) and in the sham group (147.3 ± 28.8% and 141.0 ± 21.5% of the control in PS amplitude and in fEPSP slope, respectively, *n* = 5) were significantly potentiated (*p* < 0.05). There were no significant differences in LTP and its IRP between PSs and fEPSPs in each group (Figures 2A,B and 3). Furthermore, there were no significant differences in LTPs and the IRP of PSs or fEPSPs between the normal and sham groups. The data from the sham group were used as the control for comparison.

### 3.2 Influences of septic conditions on LTP

**Figure 4:**
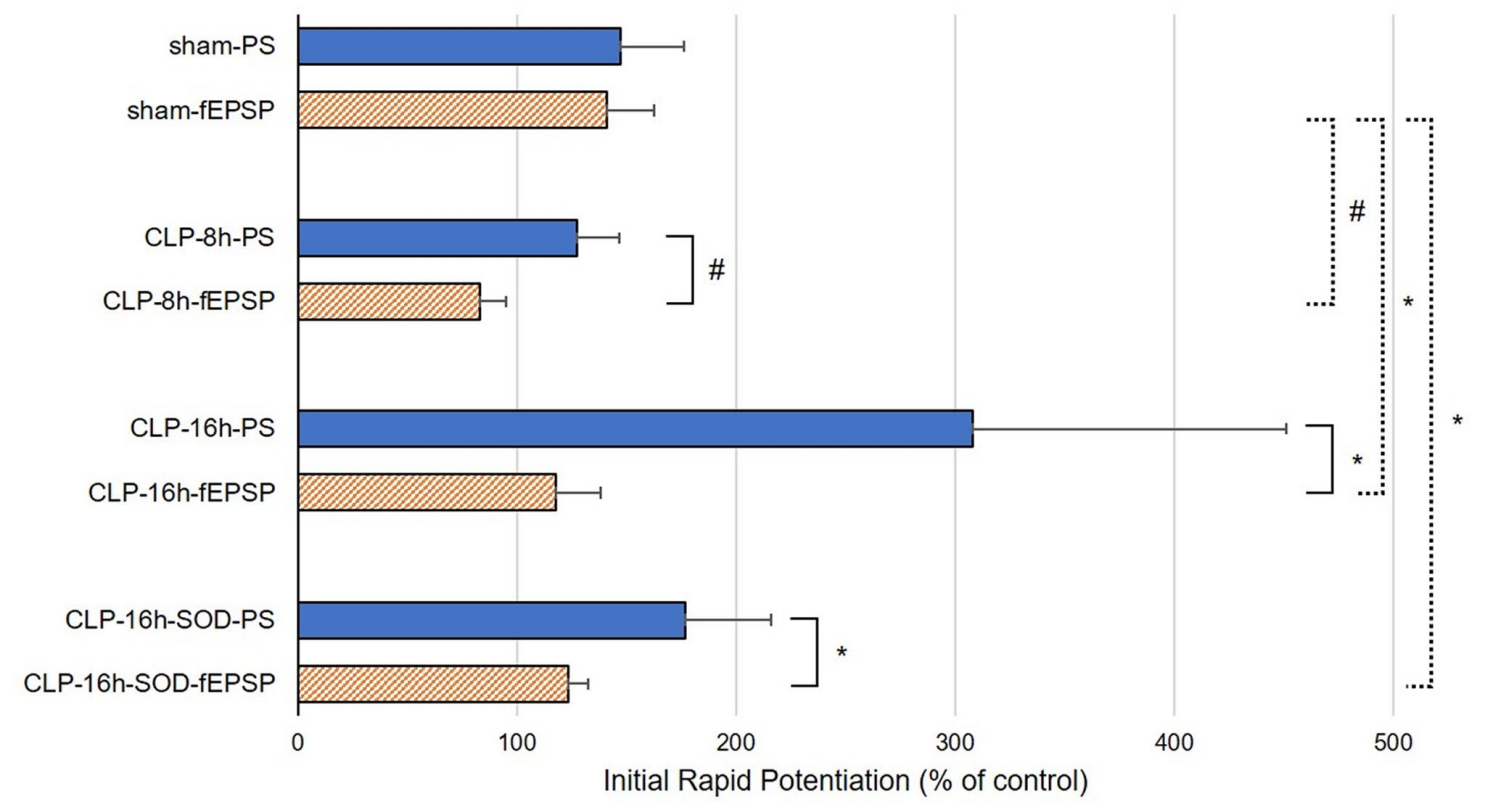
Initial rapid potentiations (IRPs) in sham, CLP-8 h, CLP-16 h, and CLP-16 h-SOD groups. There was no significant difference in IRPs between population spikes (PSs) and field excitatory postsynaptic potentials (fEPSPs) in the sham group (*n* = 5); however, there were significant differences in IRPs between PSs and fEPSPs in the CLP-8 h group (*n* = 5), the CLP-16 h group (*n* = 5), and the CLP-SOD group (*n* = 5). ^#^*p* < 0.01 between PSs and fEPSPs, *p < 0.05 between PSs and fEPSPs.

**Figure 5:**
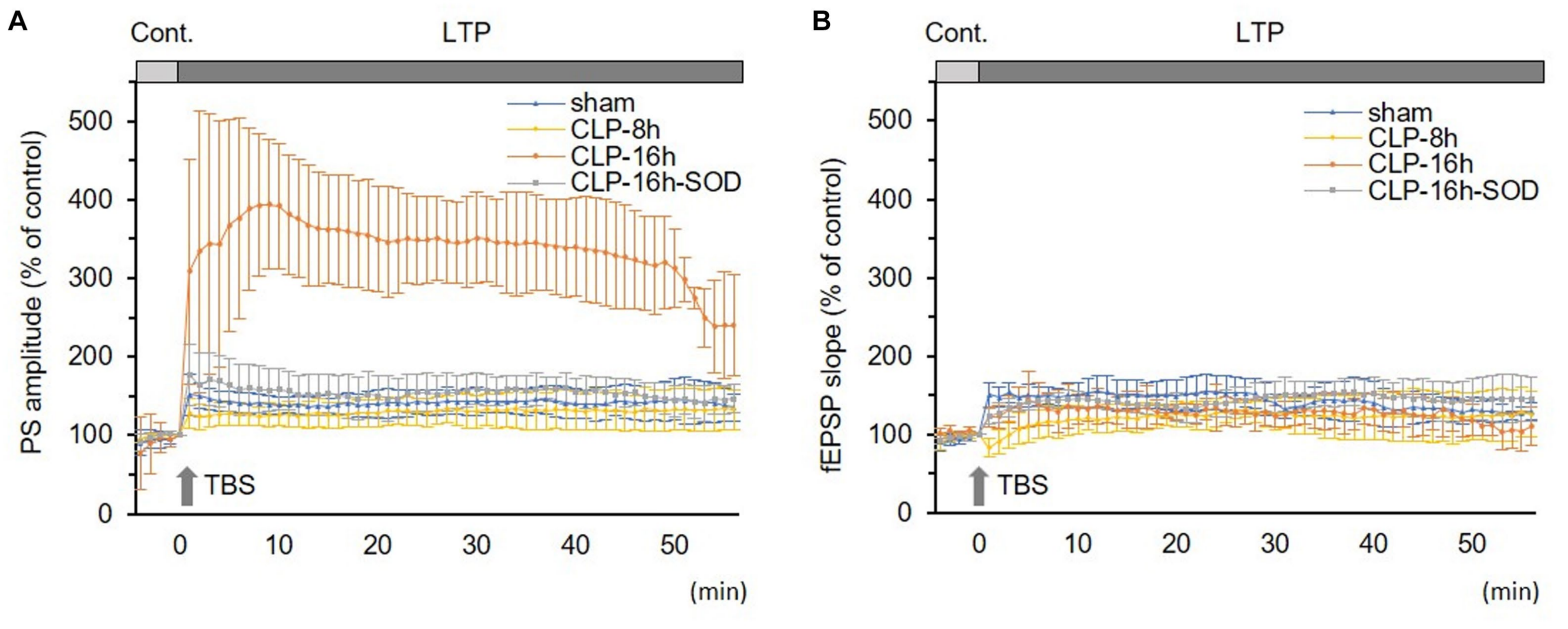
Comparisons in long-term potentiations (LTP) of population spikes (PSs, **A**) and field excitatory postsynaptic potentials (fEPSPs, **B**) among experimental groups. **(A)** LTP of PS was significantly larger in the CLP-16 h group compared to the sham, CLP-8 h, and CLP16h-SOD groups. There was no significant difference in LTPs of PSs among the sham, CLP-8 h, or CLP16h-SOD groups. **(B)** There was no significant difference in LTPs of fEPSPs among each group. Data are expressed as mean ± standard deviation and % of the control value.

In the CLP-8 h group, TBS-induced LTPs in PSs and fEPSPs (*p* < 0.05, *n* = 5, Figure 2C), and there were no significant differences between these LTPs (Figure 2C). However, IRP appeared only in PSs (127.6 ± 19.4% of the control, *p* < 0.05, Figures 3, 4). IRP in fEPSPs was not seen, but rather suppressed (83.4 ± 11.6% of the control, Figures 3, 4), and thereafter LTPs developed gradually with a delayed time course (Figure 2C). Compared with the sham group, there were no significant differences in LTPs of PSs and fEPSPs in the CLP-8 h group (Figure 5), and IRP in fEPSPs was significantly suppressed (*p* < 0.01, Figure 4). In the CLP-16 h group, TBS induced LTPs in PSs and fEPSPs (*p* < 0.05, *n* = 5, Figure 2D). The LTP of PSs was huge, its peak was delayed, and the LTP of PSs was significantly larger than that of fEPSPs (*p* < 0.01). IRP in PSs was 308.2 ± 143.0% of the control; however, that of fEPSPs was smaller (117.7 ± 20.7% of the control, Figures 3, 4). The onset and development of these LTPs were gradual. Compared with the sham group, the LTP of PSs in the CLP-16 h group was larger (*p* < 0.01); by contrast, that of fEPSPs showed no significant difference (Figure 5), and IRP in fEPSPs was significantly suppressed (*p* < 0.05, Figure 4).

### 3.3 Action of a free radical scavenger on the influence of sepsis on LTP

In the CLP-16 h-SOD group, TBS induced LTPs in PSs and fEPSPs (*p* < 0.05, *n* = 5, Figure 2E), and there were no significant differences in these LTPs (Figure 2E). The onset of LTP in PSs was rapid, but that of fEPSPs was slow and developed gradually. IRP in PSs (176.9 ± 38.9% of the control) was significantly larger than that in fEPSPs (123.3 ± 9.4% of the control, *p* < 0.05, Figures 3, 4). The LTP of PSs in the CLP-16 h-SOD group was smaller than that in the CLP-16 h group (*p* < 0.05, Figure 5). There was no significant difference in LTP of PSs and fEPSPs between the CLP-16 h-SOD and sham groups (Figure 5). IRP in fEPSPs was significantly suppressed compared with that in the sham group (*p* < 0.05, Figure 4).

## 4 Discussion

The present study demonstrates that sepsis modulates TBS-induced hippocampal LTP of PSs and fEPSPs in different manners. The results of this study highlight some important facts: (i) in early and late sepsis, the IRP component of LTP in fEPSPs was initially suppressed or reduced, then the LTP developed gradually; (ii) in late sepsis, LTP in PSs was strongly potentiated, and its peak was delayed; and (iii) the late sepsis-induced potentiation of LTP in PSs was depressed by SOD.

The dissection view in sham or CLP-operating rats supports the results of previous studies that conditions after 8 h or 16 h of CLP correspond to early and late sepsis, respectively (Chaudry et al., 1979; Chen et al., 1994). Sham laparotomy did not influence the IRPs and LTPs of the PSs and fEPSPs, indicating that the CLP-induced changes in LTPs observed in the present study are induced by sepsis.

LTP of fEPSPs and PSs induced by TBS in the hippocampal CA1 region is characterized by a sustained increase in excitatory synaptic transmissions and a concomitant enhancement of action potentials, respectively (Bliss and Lomo, 1973). In fEPSPs, IRP was depressed more intensely in the CLP-8 h group compared to the CLP-16 h group; however, whole LTPs were not different among sham and these CLP groups, demonstrating that the latter parts of the LTPs were not different among these groups. These results indicate that sepsis depresses only the initial phase of LTP of glutamatergic excitatory synaptic transmissions, and depression appears more strongly in early sepsis compared to late sepsis. In our previous study, using the neuromuscular junction (a single cholinergic excitatory synapse), it was reported that CLP-late sepsis increased quantal acetylcholine release from the nerve terminal in rats (Niiya et al., 2006). It is strongly presumed that the influence of sepsis on nerve terminals further enhances TBS-induced facilitation of glutamate release (Ghijsen et al., 1992), one of the main mechanisms of LTP; thereafter, quantal glutamate storage in nerve terminals is temporarily exhausted immediately after TBS; therefore, IRP is depressed. However, reasons why the influence of early sepsis on excitatory synaptic transmission appears more intensely than that of late sepsis are still unrevealed. For further investigation, elucidation of the influences of sepsis on other main mechanisms inducing LTP, i.e., activating N-methyl-D-aspartate (NMDA) glutamate receptors (Bliss and Lomo, 1973) and suppressing inhibitory synaptic transmission (Camire and Topolnik, 2014; Lau et al., 2017), is necessary.

In the CLP-16 h group, IRP and whole LTP in PSs were strongly potentiated, accompanied by a lack of potentiation of whole LTP in fEPSPs. In the CLP-8 h group, IRP and whole LTP in PSs and whole LTP in fEPSPs remained unchanged; however, IRP in fEPSPs was depressed. These results indicate that late sepsis facilitates LTP of action potentials generated on soma. In our previous study, it was reported that the regression of the PS-fEPSP relationship, simultaneously recorded using the MEA system, is linear in the normal rat hippocampus (Narimatsu et al., 2013); therefore, the divergences between PSs and fEPSPs suggest an increase in somatic excitability. It has been reported in our previous study using the neuromuscular junction that CLP-late sepsis decreased critical depolarization on the postsynaptic membrane (Niiya et al., 2006). These strongly suggest that sepsis potentiates somatic excitability by decreasing critical depolarization, and the potentiation progresses stage-dependently. The delay in peak LTP in PSs in the CLP-16 h group seems like a reflection of that in fEPSPs. For further investigation, elucidation of the influences of sepsis on GABA-ergic inhibitory synaptic transmission and modulating postsynaptic excitability (Abraham et al., 1987) is required.

IRPs and whole LTPs of PSs and fEPSPs in the CLP-16 h-SOD group were not different from those in the sham group. Strongly potentiated LTP of PS, observed in the CLP-16 h group, disappeared in the CLP-16 h-SOD group. These results indicate that SOD, an antioxidant enzyme, suppresses the influences of late sepsis, i.e., potentiation in LTP of somatic action potentials. SOD removes superoxide anions and converts them into oxygen and H_2_O_2_; thereafter, the H_2_O_2_ is subsequently metabolized by catalase and peroxidase (Berg et al., 2011). If some ROS other than superoxide were significantly involved in the mechanisms, SOD alone should not perform such an almost complete inhibitory action. Therefore, superoxide is probably the main cause of the effects of late sepsis. The mechanisms underlying septic brain dysfunction are not yet fully understood; however, the participation of ROS has been reported (Bozza et al., 2013). Increased superoxide is reported in the brain stem of septic rats (Barichello et al., 2006; Ninković et al., 2006). On the other hand, superoxide is physiologically required for the induction of LTP, as shown by the inhibition of LTP in fEPSPs in the hippocampal CA1 region by cell-impermeable superoxide scavengers (Klann et al., 1998). It is strongly suspected that excessive superoxide, overproduced by sepsis, intensely potentiates somatic excitability in late sepsis. Effects of superoxide on the firing of pyramidal cells and on GABA-ergic inhibitory synaptic transmissions are the topics for future studies.

This study has some limitations. First, histological assessments were not performed in CLP-induced groups, potentially resulting in individual differences in the degree of brain edema or neuroinflammation. Second, PEG-SOD in the brain was not measured. A previous study reported no major uptake of PEG-SOD in the brains or cerebrospinal fluid of normal rats (Yoshida et al., 1992). However, it is suggested that hypertension or sepsis can increase vascular permeability (Yoshida et al., 1992; Erikson et al., 2020). Therefore, the distribution of PEG-SOD in the brains of normal and septic animals should be considered and tested in future work.

In the groups where CLP was performed, hippocampal slices were prepared from brain hemispheres removed from septic rats and consequently incubated in normal artificial ACSF; however, septic changes in PSs and fEPSPs were recorded from these slices. These demonstrate that the influences of sepsis observed in the present study do not depend on some septic factors dissolved in extracellular fluid but on sepsis-induced long-term modulations or damages to the functions of the CNS. Therefore, the results obtained in the present study reflect the pathophysiological synaptic changes in persisting clinical SAE after recovery from a septic condition. Furthermore, regulation of excessive superoxide in the CNS using free radical scavengers could be effective for clinical SAE.

## 5 Conclusion

The rapid potentiation of LTP in fEPSPs was suppressed or reduced in the early septic models. In the late septic model, LTP in PSs was greatly increased, with a delayed peak. SOD depressed the influence of late sepsis. These results indicate that septic excessive superoxide generates sepsis-induced changes in the LTPs. The findings in the present study are probably the key mechanisms inducing clinical SAE.

## Data availability statement

The raw data supporting the conclusions of this article will be made available by the authors, without undue reservation.

## Ethics statement

The animal study was approved by the Animal Use Committee of Sapporo Medical University. The study was conducted in accordance with the local legislation and institutional requirements.

## Author contributions

RE: Writing – original draft, Data curation, Formal analysis, Investigation. EN: Writing – review & editing, Conceptualization, Methodology, Supervision. TK: Writing – review & editing. KN: Writing – review & editing.
